# Knowledge, attitudes and peer influences related to pregnancy, sexual and reproductive health among adolescents using maternal health services in Ugu, KwaZulu-Natal, South Africa

**DOI:** 10.1186/s12889-019-7242-y

**Published:** 2019-07-11

**Authors:** Desiree Govender, Saloshni Naidoo, Myra Taylor

**Affiliations:** 1 KwaZulu-Natal Department of Health, Durban, South Africa; 2 School of Nursing and Public Health, Discipline of Public Health Medicine, University of KwaZulu-Natal, Durban, South Africa; 3 Developing Research Innovation Localisation and Leadership (DRILL) Fellow, University of KwaZulu- Natal, Durban, South Africa

**Keywords:** Adolescent pregnancy, HIV, Sexually transmitted infections, Safe motherhood initiative, Sexual and reproductive health

## Abstract

**Background:**

Knowledge and practices of sexual and reproductive healthcare is pivotal to the Safe Motherhood Initiative; however, only a few studies have investigated adolescent mothers’ knowledge of sexual and reproductive health in light of the above initiative. Research should thus focus on the knowledge and attitudes of adolescent girls as well as peer influences related to pregnancy and sexual and reproductive health among adolescents, as the findings may highlight vital health interventions that should be introduced. The aim of this study was thus to determine the knowledge, personal attitudes and peer influences related to pregnancy, sexual and reproductive health among adolescents who attended maternal health services in a district hospital in Ugu, KwaZulu-Natal, South Africa.

**Methods:**

A cross sectional study was conducted. Data were collected from 326 adolescents who accessed maternal health services in a peri-urban district hospital during June 2017 and November 2017. The questionnaire surveyed the knowledge, personal attitudes and peer influences related to pregnancy, sexual and reproductive health. The questionnaire was administered by fieldworkers using mobile devices powered by the Mobenzi Researcher® technology. The completed surveys were uploaded to the Mobenzi server where it was stored and aggregated. The data was analysed using R software.

**Results:**

Of the 326 participants, 65 (19.9%) experienced repeat pregnancies in adolescence. Overall, only 143 (43.9%) of the participants answered 50% or more of the knowledge questions on pregnancy and HIV/AIDS and STIs correctly, while 183 (56.1%) answered less than 50% of the knowledge questions correctly. There was no relationship between knowledge of pregnancy and HIV/STIs and repeat adolescent pregnancies.

**Conclusion:**

Adolescents’ knowledge of pregnancy and sexual and reproductive health was deficient as, even with repeat pregnancies, these adolescents were evidently no better informed about pregnancy and sexual and reproductive health. This suggests that social determinants, modes and platforms regarding the delivery of adolescent sexual and reproductive health education are important. An innovative mode to the delivery of sexual and reproductive health education includes the emerging digital platform. The digital platform encompasses social media, multimedia and mobile phones which is growing popular among young people.

**Electronic supplementary material:**

The online version of this article (10.1186/s12889-019-7242-y) contains supplementary material, which is available to authorized users.

## Background

Adolescence is a complex stage in life that is characterised by conflicts among responsibilities, independence and experimentation [1], and health and social problems may be extensive during this phase [2]. According to the World Health Organization, the main health issues among adolescents include early pregnancy, childbirth, HIV/AIDS, depression, violence, alcohol and drug abuse, intentional injuries, malnutrition, obesity, and tobacco use [3]. Adolescents account for 1.2 billion people globally and 11% of all births worldwide are to girls aged 15–19 years [4]. South Africa is home to approximately 9.68 million adolescents [5] and the adolescent pregnancy rate here is 47 births per 1000 females aged 15–19 per annum [6]. Adolescent pregnancy and childbearing is increasingly recognised as a serious, worldwide public health concern. The maternal mortality rate associated with pregnant adolescents has been attributed to pregnancy induced hypertension, HIV/AIDS, Tuberculosis, obstetric haemorrhaging, and medical and surgical disorders [6, 7]. The HIV prevalence in South Africa is estimated at 13.1% [5].

Sexual and reproductive health (SRH) programs are often not prioritised due to a lack of funding and the emphasis that is placed on community-based HIV prevention programmes [8]. There are also restrictive laws and policies concerning adolescent sexual and reproductive health [9]. Access to SRH in the Asia Pacific Region is dependent on age and the regulation of marital status [10, 11]. Customary and religious laws require persons under the age of 18 years to seek consent from parents or spouses to access SRH [10]. Healthcare providers may also be prohibited from delivering contraceptive services to unmarried persons [11]. The age that an adolescent is allowed to consent for sex often conflicts with the age that the adolescent is allowed to consent for medical interventions [10]. Thus, the blatant disregard of adolescent SRH often results in a damaging transition to adulthood [9]. Education and clinical services, which are the cornerstones of SRH, are essential in promoting positive sexual development and decreasing adverse social, economic and health outcomes associated with sexual behaviour [12]. Parents, teachers, healthcare providers and community leaders are the gatekeepers of adolescent sexual and reproductive health because they are responsible sources of SRH information and services for adolescents [13].

The Safe Motherhood Initiative was a key outcome of the first international Safe Motherhood Conference in Nairobi, Kenya, in 1987 [14]. The pillars of the Safe Motherhood Initiative include family planning, antenatal care, obstetric care, postnatal care, post abortion care and STI/HIV/AIDS control. The Safe Motherhood Initiative is founded on the principle of sound knowledge and practices of reproductive health; however, only a few studies have investigated adolescent mothers’ knowledge of sexual and reproductive health [14]. Universal access to SRH services is one of the specific targets of the Sustainable Development Goals pertaining to health (SDG3) [15]. Thus knowledge, personal attitude and peer influences can influence adolescents’ decision to access maternal, sexual and reproductive health services or not. However, far too little attention has been paid to the knowledge, personal attitudes and peer influences related to pregnancy, sexual and reproductive health among adolescent girls accessing maternal health services.

A lack of pregnancy related knowledge among pregnant adolescent girls can adversely affect their lives as well as those of their unborn children [16]. Therefore, adolescent pregnant women should be equipped with knowledge so that they will be able to engage in good health practices during pregnancy. They should also identify danger signs during pregnancy and make prompt decisions about appropriate care and support. The age and maturity level of pregnant women may impact their susceptibility to antenatal education and their ability to identify danger signs associated with their pregnancy [17]. However, knowledge of SRH and pregnancy among adolescents has remained inadequate [4], and therefore adolescent pregnancy is considered a high health risk as poor nutrition, limited knowledge, and ignorance of danger signs during pregnancy can be devastating.

This paper is an excerpt from a larger doctoral study that aims to develop a community of practice model for a multidisciplinary and comprehensive approach towards caring for pregnant and parenting adolescent mothers. This doctoral study is being conducted in Ugu, KwaZulu-Natal, South Africa. The purpose of this paper is to report the knowledge, personal attitudes and peer influences related to pregnancy, sexual and reproductive health among adolescents who utilised maternal health services in a district hospital in Ugu, KwaZulu-Natal, South Africa.

## Methods

### Study design and setting

A descriptive cross-sectional study design was used to assess the knowledge, attitudes and peer influences related to pregnancy, sexual and reproductive health among adolescents using maternal health services in the study site during June 2017 to November 2017. The target sample size of 326 participants were reached for the study. The study was conducted in a district hospital in Ugu, KwaZulu Natal. At the time of the study, the majority of the study population (84%) resided in rural areas whereas only 16% resided in the urban coastal strip. Generally, KwaZulu-Natal has the highest prevalence of HIV in pregnant women (44.4, 95% CI: 42.5–46.3%) among the South African provinces [18]. According to the South African National Antenatal Sentinel HIV and Syphilis Prevalence Survey, HIV prevalence is 45.9% in the Ugu district [18].

### Study population and sampling

The study population included adolescent girls aged 13–19 years manifesting first and repeat pregnancies and accessing the maternal health services at the district hospital. Convenience sampling was used to recruit these females. Adolescents with first and repeat pregnancies who attended the ante- and postnatal clinics were identified by the nurses and briefly informed about the study. The nursing staff was aware of the inclusion and exclusion criteria, which included the adolescents’ cognitive abilities, and they recruited participants accordingly. The nursing staff used the questions from the modified mini mental state exam (MMSE) to determine the participants cognitive function. The MMSE is a widely used measure of global cognitive function [19]. The questions in the MMSE with regards to the tests of orientation (time, place, person), registration and recall are used in the assessment of the maternity healthcare users at the institution. Interested participants were referred to the research assistants stationed in the clinic who then provided them with more details, after which they decided whether to participate voluntarily or not.

The sample size of 326 participants were determined for the study on the prevalence of adolescent repeat pregnancy at the study site. Details of the sample size calculation for the prevalence study has been described elsewhere. The eligibility criteria for participants were: 1) pregnant and postpartum adolescent girls aged 13–19 years, and 2) pregnant and postpartum adolescents under 18 years (but above 13 years) who had obtained permission from their parent/s or legal guardian/s to participate in the study. The exclusion criteria included: 1) pregnant and postpartum adolescents under 13 years of age; 2) pregnant and postpartum adolescents under 18 years of age who had not obtained permission from their parent/s or legal guardians to participate in the study; and 3) pregnant and postpartum adolescents with cognitive impairment. Parental or legal guardian consent for participants under the age of 18 years was stipulated by the Biomedical Research Ethics Committee, UKZN.

### Measurement instrument

The Healthy Pregnancy Knowledge Survey (HPKS) questionnaire, which was developed by Godin et al. [20], was adapted for this study to measure the participants’ knowledge related to pregnancy health. The HPKS questionnaire contained 25 questions relating to three major topics: healthy pregnancy, a healthy lifestyle, and breastfeeding. The questionnaire was originally piloted in thirty public health institutions in Ontario. Although the validity and reliability of the HPKS were not formally assessed, professionals and academics in the fields of public health and maternal and child health gave relevant and comprehensive input for the development of the HPKS. The questionnaire contained multiple choice questions and a ‘true’ and ‘false’ scale with a ‘do not know’ option. Knowledge and practices of sexual and reproductive healthcare included questions on: 1) HIV/AIDS and STIs; 2) sexual risk behaviour and prevention; 3) personal attitudes towards sexuality and reproduction; and 4) peer influences.

The questionnaire also included questions about topics that were taught in the Life Orientation curriculum at school (Additional file 1). A Life Skills programme was introduced in secondary schools in South Africa in 1996 to provide learners with comprehensive information on HIV/AIDS and STIs, reproduction, contraception, pregnancy, violence, decision making and sexual negotiation [21]. This programme has now been integrated into the Life Orientation curriculum. Life Orientation is a standardised subject in school that focuses on topics such as teenage pregnancy, contraception, STIs, HIV/AIDS, sexuality, and reproduction.

The South African Department of Basic Education [22] describes Life Orientation as “the study of the self in relation to others and to society. It addresses skills, knowledge and values about the self, the environment, responsible citizenship, a healthy and productive life, social engagement, recreation and physical activity, careers and career choices” (p.8). Life Orientation is an essential subject required for the National Senior Certificate and is thus a compulsory subject for all learners in Grades 10, 11 and 12 in South Africa.

The questionnaire was piloted among 25 pregnant adolescents by fieldworkers using mobile devices powered by Mobenzi Researcher® technology. The study site was the antenatal unit at the hospital where the current doctoral study is conducted. The aim of a pilot study was to establish the user friendliness of the questionnaire. A gynaecologist, a clinical midwife, a school health manager, a clinical psychologist and an adolescent and reproductive academic expert were consulted to establish the face and content validity of the questionnaire.

### Data collection

Fieldworkers were recruited from the local community and received training on research ethics, the aims and objectives of the study, and mobile data collection. The questionnaire survey (measurement instrument) was administered by the fieldworkers using mobile cellular tablets powered by the Mobenzi Researcher® technology. The responses to the survey were obtained in mobile assisted face-to-face interviews between the fieldworkers and individual female adolescents. The interviews were conducted in safe, private settings within the ante- and postnatal units. Mobenzi Researcher®, which was developed in 2008 in South Africa, is a platform for the mobile collection of baseline data. The software was installed on the handsets. The mobile application technology supports various question types and also allows for the marking of fields as ‘mandatory’ and ‘not applicable’. The completed surveys were uploaded to the Mobenzi survey where they were stored and aggregated.

### Data analysis

The data from the research console were cleaned and exported into R software (R version 3.5.0. Vienna: R Foundation for Statistical Computing) for statistical analyses. Frequency distributions and summary statistics were used to describe the data. The percentage of adolescent girls who were able to answer 50% of the questions about pregnancy and HIV/AIDS/STI knowledge correctly was determined. A study conducted in Malaysia had used 50% as a cut-off score for reasonable knowledge of pregnancy and sexual and reproductive health [16]. The Fisher’s exact test was used to ascertain statistical significance of categorical variables while the Wilcoxon signed ranked test was used to perform comparisons between continuous variables. A univariate logistic regression model was constructed with adolescent repeat pregnancy as the outcome variable of interest. The main exposure of interest was whether or not the adolescent girls answered 50% of the pregnancy and HIV/AIDS/STI related questions correctly. A multivariate logistic regression model was created similar to the univariate logistic regression model, but it adjusted for age, education levels, and the mothers’ marital status. A *p*-value of 0.05 was considered statistically significant.

## Results

### Socio-demographic, obstetric, clinical characteristics and knowledge survey results

A total of 326 participants completed the questionnaire. The majority of the participants was in the age group 17–19 years (86.5%, *n* = 282). Of the 326 participants, 98.5% was single (Table 1). All the participants indicated that they had received formal education while 51.5% (*n* = 168) indicated that their biological mothers had received formal education. Most of the participants (97.5%, *n* = 318) were unemployed. Only 102 (31.0%) of the participants indicated that their biological mothers were married, while 147 (45.1%) stated that their biological mothers were single.

**Table 1:** Socio-demographic, obstetric, clinical characteristics and knowledge survey results (*n* = 326)

Variables	Frequencies (n)	Percentages (%)
Age		
14–16	44	13.5%
17–19	282	86.5%
Marital status of participants		
Single	321	98.5%
Married	5	1.5%
Highest educational level		
Primary	9	2.8%
Junior	12	3.7%
Secondary	305	93.6%
Employment status of participants		
Employed full time	6	1.8%
Employed part time	2	0.6%
Unemployed	318	97.5%
Family characteristics		
Biological mother’s marital status		
Single	147	45.1%
Married	101	31%
Other	78	23.9%
Educational level of biological mother		
Primary school	29	8.9%
Junior secondary	17	5.2%
Senior secondary	118	35.6%
Technical school	3	0.9%
University degree	3	0.9%
None	28	8.6%
Do not know	130	39.9%
Obstetric history		
Had an adolescent repeat pregnancy?		
No	261	80.1%
Yes	65	19.9%
Number of spontaneous abortions		
0	298	91.4%
1	27	8.3%
2	1	0.3%
Medical conditions		
High blood pressure	23	7.1%
Anaemia	25	7.7%
HIV	58	17.8
Answered knowledge questions correctly (Pregnancy, HIV/AIDS and STIs)		
50% or more questions correct	143	43.9%
Less than 50% correct	183	56.1%

Of the 326 participants, 65 (19.9%) had experienced repeat pregnancies during their adolescent years and 28 (8.6%) of the participants admitted that they had experienced a spontaneous abortion. The clinical histories revealed that 23 (7.1%) of the participants had experienced pregnancy induced hypertension while 25 (7.7%) had experienced anaemia. The prevalence of HIV among the participants was 17.8% (*n* = 58).

### Life orientation

The participants were asked if HIV/AIDS, STIs, contraception, menstruation, male circumcision, teenage pregnancy, termination of pregnancy, abuse, depression and anxiety were covered in the Life Orientation curriculum when they attended school. Of the 326 participants, 129 (39.6%) stated that contraception was not covered in Life Orientation. Furthermore, 154 (47.2%) had not learnt about the termination of pregnancy in Life Orientation. Most participants, 77.9 and 72.9% respectively, stated that HIV/AIDS and STIs was covered in Life Orientation. When asked about adolescent pregnancy, 286 (87.7%) reported that this topic was covered in Life Orientation while only 164 (50.3%) reported that depression and anxiety were covered in the Life Orientation curriculum at school.

### Miscellaneous general pregnancy and signs of preterm labour knowledge

A total of 303 (92.9%) correctly identified that a pregnant woman must consult her clinician (doctor or midwife/nurse) should she feel unwell, has a fever, and/or experiences an unusual physical change (Table 2). Less than half of the participants (44.2%, *n* = 144) was able to correctly identify the advice that pregnant women should follow to decrease the risk of preterm labour. Only 116 (35.6%) knew that folic acid is essential for the baby’s brain and spine development. The most commonly identified signs of preterm labour were understood as cramps and abdominal pain (48.8%, *n* = 159), the release of fluid or blood from the vagina (60.4%, *n* = 197), and regular or frequent contractions or changes in the severity or number of contractions (33.7%, *n* = 110) (Table 2). When questioned about what a pregnant woman should do in the event of preterm labour, most participants were able to correctly identify that a pregnant woman should contact the doctor (87.7%, *n* = 286) or go to the hospital immediately (94.2%, *n* = 307).

**Table 2:** General knowledge pertaining to pregnancy and signs of preterm labour (*N* = 326)

Questions	Correct Answer	Frequencies (n)	Percentages (%)
A pregnant woman must consult a clinician (doctor or midwife/nurse)	True		
True		303	92.9%
False		2	0.6%
Do not know		21	6.4%
A pregnant woman must consult her clinician (doctor or midwife/nurse) in the event that she feels unwell, has a fever, and/or experiences an unusual physical change.	True		
True		301	92.3%
False		2	0.6%
Do not know		23	7.1%
To decrease the risk of preterm labour, a pregnant woman should:	All of the above		
Attend antenatal care as soon as she knows she is pregnant		79	24.2%
Reduce stress		20	6.1%
Follow all the healthcare advice given by the doctor or midwife		15	4.6%
Eat healthy, nutritious food		4	1.2%
All of the above		144	44.2%
Do not know		64	19.6%
Folic acid is taken by pregnant women because it:	Is essential for the baby’s brain and spine development		
Prevents nausea		6	1.8%
Is essential for the baby’s brain and spine development		116	35.6%
Prevents sexually transmitted infections		2	0.6%
Helps with the baby’s lung development		6	1.8%
Helps to reduce lower back pain		1	0.3%
Do not know		195	59.8%
Pregnant women should avoid exercise.	False		
True		63	19.3%
False		210	64.4%
Do not know		53	16.3%
Signs of preterm labour			
Persistent cramps and abdominal (stomach pain)	Yes		
Yes		159	48.8%
No		167	51.2%
The release of fluid or blood from the vagina	Yes		
Yes		197	60.4%
No		129	39.6%
Regular or frequent contractions or changes in the strength or number of contractions	Yes		
Yes		110	33.7%
No		216	66.3%
A feeling as if the baby is pushing down	Yes		
Yes		99	30.4%
No		227	69.6%
Swollen ankles	No		
Yes		24	7.4%
No		302	92.6%
Persistent cramps and abdominal (stomach pain)	Yes		
Yes		159	48.8%
No		167	51.2%

### Normal symptoms during pregnancy

A total of 235 (72.1%) correctly identified that aches in the hips and back are a normal symptom during pregnancy. The results showed that 19 (5.8%) of the participants did not know that nausea was experienced during pregnancy. Only 143 (43.9%) and 76 (23.3%) correctly identified that an abnormal vaginal discharge and spotted vision were abnormal symptoms during pregnancy respectively.

### Knowledge of anaemia

Most participants (63.2%, *n* = 206) did not know that iron deficiency leads to anaemia. Less than half (43.3%, *n* = 141) of the participants knew that anaemia can result in preterm delivery and low birth weight. When asked to identify the signs and symptoms of anaemia, only 15 (4.6%) were able to identify six out of thirteen signs and symptoms of anaemia. The majority of the participants (59.5%, *n* = 194) was able to identify only one out of thirteen signs and symptoms of anaemia. The most commonly identified signs and symptoms of anaemia were pale skin (46.3%, *n* = 151), pale lips (31.3%, *n* = 102) and pale underside of the eyelids (31%, *n* = 101).

### Knowledge of alcohol and tobacco use during pregnancy

When assessing knowledge of alcohol use during pregnancy, most of the participants (92.5%, *n* = 302) knew that no quantity of alcohol is safe during pregnancy (Table 3). Only 64 (19.6%) and 67 (20.6%) agreed that alcohol consumption results in low birth weight and heart defects respectively. Of the 326 participants, 209 (64.1%) knew that alcohol consumption can cause mental retardation in the unborn child. Few participants knew that tobacco use during pregnancy results in low birth weight (22.4%, *n* = 73), spontaneous abortion (16.6%, *n* = 54), preterm labour (6.7%, *n* = 22), and foetal deformities in the uterus (16%, n-52).

**Table 3:** Knowledge regarding alcohol and tobacco use during pregnancy (*N* = 326)

Questions	Correct Answer	Frequencies (n)	Percentages (%)
What quantity of alcohol may a pregnant woman consume?	No quantity of alcohol is safe during pregnancy.		
1 alcoholic drink a day		1	0.3%
No quantity of alcohol is safe during pregnancy.		302	92.6%
Do not know		23	2.1%
Alcohol consumption during pregnancy results in low birth weight.	Yes		
Yes		64	19.6%
No		262	80.4%
Alcohol consumption during pregnancy results in heart defects.	Yes		
Yes		67	20.6%
No		259	79.4%
Alcohol consumption during pregnancy results in damage to the unborn baby’s liver.	Yes		
Yes		118	36.2%
No		208	63.8%
Alcohol consumption during pregnancy results in mental retardation in the unborn baby.	Yes		
Yes		209	64.1%
No		117	35.9%
Smoking tobacco during pregnancy results in low birth weight.	Yes		
Yes		73	22.4%
No		253	77.6%
Smoking tobacco during pregnancy results in miscarriage.	Yes		
Yes		54	16.6%
No		272	83.4%
Smoking tobacco during pregnancy results in preterm labour.	Yes		
Yes		22	6.7%
No		304	93.3%
Smoking tobacco during pregnancy results in deformities in the unborn baby.	Yes		
Yes		52	16%
No		274	84%
	No		
Smoking tobacco during pregnancy reduces back pain.		2	0.6%
Yes		2	0.6%
No		324	99.4%

### Knowledge of activities that should be avoided during pregnancy

Less than half of the participants (42.9%, *n* = 140) knew that the use of pesticides should be avoided during pregnancy. Only 41.1% (134) agreed that playing with cats is dangerous and 37% (121) knew that cleaning a cat’s litter box should be avoided during pregnancy. Only 20 (6.1%) of the participants knew that X-rays should be avoided during pregnancy.

### Knowledge regarding breastfeeding

Most participants (71.8%, *n* = 234) identified that 6 months is the recommended length of time for exclusive breastfeeding (Table 4). When assessing their knowledge about the benefits of breastfeeding, most participants correctly agreed that breast feeding improves the immunity of babies (78.8%, *n* = 257), decreases the risk of obesity and diabetes (58.3%, *n* = 190), and that breast-fed babies have fewer dental cavities (59.2%, *n* = 193). Furthermore, 200 (61.3%) knew that drugs and alcohol pass through breast milk.

**Table 4:** Knowledge related to breastfeeding (*N* = 326)

Questions	Correct Answer	Frequencies (n)	Percentages (%)
Exclusive breastfeeding is recommended for:	6 months		
6 weeks		3	0.9%
2 months		1	0.3%
6 months		234	71.8%
8 months		13	4%
Don’t know		75	23%
Breastfeeding improves the immunity of the baby and protects the baby from allergies and asthma.	True		
True		257	78.8%
False		8	2.5%
Do not know		61	18.7%
A breast-fed baby has a lower risk of obesity and diabetes.	True		
True		190	58.3%
False		23	7.1%
Do not know		113	34.7%
Breastfeeding is more time consuming than bottle feeding.	False		
True		130	39.3%
False		68	20.9%
Do not know		128	39.3%
Breast-fed babies have fewer dental cavities (i.e., problems with tooth decay).	True		
True		193	59.2%
False		31	9.5%
Do not know		102	31.3%
Drugs and alcohol can be passed through breast milk to the baby.	True		
True		200	61.3%
False		25	7.7%_
Do not know		101	31%

### Knowledge of HIV/AIDS and STIs

Only 72 (22.1%) of the participants knew that HIV can be transmitted via oral sex and 67 (20.6%) knew that anal sex transmits HIV (Table 5). Most participants correctly agreed that HIV infects and damages the CD4 cells (79.4%, *n* = 259) and that HIV develops into AIDS (81.6%, *n* = 266). Furthermore, 235 (72.1%) knew that HIV is present in semen, blood, vaginal secretions and breast milk. When asked to identify the signs and symptoms related to AIDS, overall, a large percentage of the participants was able to identify rapid weight loss (80.1%, *n* = 261), extreme weakness (75.2%, *n* = 245), chronic diarrhoea (62.6%, *n* = 204), white sores in the mouth (76.4%, *n* = 249), and sores in the genital region (66%, *n* = 215) as indicators.

**Table 5:** Knowledge regarding HIV/AIDS and STIs (*N* = 326)

Questions	Correct Answer	Frequencies (n)	Percentages (%)
HIV is known as Human Immunodeficiency Virus.	True		
True		266	81.6%
False		5	1.5%
Do not know		55	16.9%
HIV can be transmitted through oral sex.	Yes		
Yes		72	22.1%
No		254	77.9%
HIV can be transmitted through vaginal sex.	Yes		
Yes		233	71.5%
No		93	28.5%
HIV can be transmitted through anal sex.	Yes		
Yes		67	20.6%
No		259	79.4%
HIV infects and damages CD4 helper cells.	True		
True		259	79.4%
False		1	0.3%
Do not know		66	20.2%
HIV develops into AIDS.	True		
True		266	81.6%
False		3	0.9%
Do not know		57	17.5%
HIV is present in semen, blood, vaginal secretions and breast milk.	True		
True		235	72.1%
False		6	1.8%
Do not know		85	6.1%
Gonorrhoea is a sexually transmitted infection.	Yes		
Yes		80	24.5%
No		246	75.5%
Syphilis is a sexually transmitted infection.	Yes		
Yes		49	15%
No		277	85%
Chlymydia is a sexually transmitted infection.	Yes		
Yes		12	3.7%
No		314	96.3%
Genital Herpes is a sexually transmitted infection.	Yes		
Yes		51	15.6%
No		275	84.4%
Genital sores are associated with the signs and symptoms of sexually transmitted infections.	Yes		
Yes		186	57.1%
No		140	42.9%
Burning during urination is associated with the signs and symptoms of sexually transmitted infections.	Yes		
Yes		179	54.9%
No		147	45.1%
Discharge from the vagina is associated with the signs and symptoms of sexually transmitted infections.	Yes		
Yes		214	65.6%
No		112	34.4%
Discharge from the penis is associated with the signs and symptoms of sexually transmitted infections.	Yes		
Yes		195	59.8%
No		131	40.2%

A low percentage of participants was able to identify Gonorrhoea (24.5%, *n* = 80), Syphilis (15%, *n* = 49), Chlamydia (3.7%, *n* = 12) and Genital Herpes (15.6%, *n* = 51) as STIs (Table 5). However, when assessing the knowledge of the signs and symptoms of STIs, most participants were able to correctly identify genital sores (57.1%, *n* = 186), burning urination (54.9%, *n* = 179), discharge from the vagina (65.6%, *n* = 214), and discharge from the penis (59.8%, *n* = 195) as symptoms.

The common prevention methods for HIV and STIs that were identified included condom use (92.6%, *n* = 302), being faithful to one partner (63.8%, *n* = 208), and refusal to share needles (67.2%, *n* = 219). Only 163 (50%) agreed that abstaining from sexual intercourse can prevent HIV and STIs. Twenty-five (7.7%) participants incorrectly thought that birth control pills can prevent HIV and STIs. One hundred and five (66%) of the participants correctly agreed that all sexually active women must have an annual pap smear. Most participants (70.2%, *n* = 229) knew that a pregnant woman who is infected with HIV and STIs can transmit these diseases to her unborn child.

### Personal attitudes towards sexuality and reproductive health

Of the 326 participants, 79 (24.2%) agreed that sex before marriage is acceptable whereas 87 (26.7%) agreed that abstaining from sex is difficult during adolescence (Table 6). Only sixty-one (18.7%) thought that a female who remains a virgin during her adolescent years is old- fashioned. Fifty- three (16.3%) agreed that using a condom during sexual intercourse reduces sexual pleasure, while 101 (30%) agreed that the use of contraceptives causes sterility in women. Furthermore, 79 (23.9%) agreed that they would be too embarrassed to buy or procure condoms and 52 (16%) agreed that their partners would reject them if they asked them to use a condom. The majority of the participants (86.8%, *n* = 283) agreed that family planning services can help prevent an unwanted pregnancy. Overall, 216 (66.3%) agreed that females are responsible for protection during sexual intercourse.

**Table 6:** Personal attitudes and peer influences regarding sexuality and reproductive health (*N* = 326)

Personal attitude statements	Frequencies (n)	Percentages (%)
Sex before marriage is acceptable.		
Agree	79	24.2%
Disagree	233	71.5%
Strongly disagree	14	4.3%
For a woman, having multiple sex partners is an indication of her attractiveness.		
Agree	10	3.1%
Disagree	240	73.6%
Strongly disagree	76	23.3%
It is important for a man to have multiple sex partners to prove his manhood.		
Agree	6	1.8%
Strongly Agree	1	0.3%
Disagree	142	43.6%
Strongly disagree	177	54.3%
A female who remains a virgin during her adolescence is old-fashioned.		
Agree	61	18.7%
Strongly agree	1	0.3%
Disagree	242	74.2%
Strongly disagree	22	6.7%
Having a baby at an early age is a sign of maturity.		
Agree	43	13.2%
Strongly agree	1	0.3%
Disagree	272	83.4%
Strongly disagree	10	3.1%
Abstaining from sex is difficult during adolescence.		
Agree	87	26.7%
Strongly agree	2	0.6%
Disagree	236	72.4%
Strongly disagree	1	0.3%
Using a condom during sexual intercourse reduces sexual pleasure.		
Agree	53	16.3%
Strongly agree	2	0.6%
Disagree	265	265 (81.3%)
Strongly disagree	6	6 (1.8%)
The use of contraceptives cause sterility in women.		
Agree	101	31%
Strongly agree	1	0.3%
Disagree	221	67.8%
Strongly disagree	3	0.9%
Family planning services can prevent an unwanted pregnancy.		
Agree	283	86.8%
Strongly Agree	4	1.2%
Disagree	39	12%
I would be too embarrassed to buy or find condoms.		
Agree	78	23.9%
Strongly agree	3	0.9%
Disagree	240	73.6%
Strongly disagree	5	1.5%
My partner would reject me if I asked him to use a condom.		
Agree	53	16%
Strongly agree	1	0.3%
Disagree	267	81.9%
Strongly disagree	6	1.8%
Females are responsible for protection during sexual intercourse.		
Agree	216	66.3%
Strongly agree	2	0.6%
Disagree	106	32.5%
Strongly disagree	2	0.6%
Peer influence statements	Response (n)	Percentage (%)
Most of my friends believe in waiting for marriage to have sex.		
Agree	76	23.3%
Strongly agree	1	0.3%
Disagree	231	70.9%
Strongly disagree	18	5.5%
Most of my friends do not believe in using contraception.		
Agree	144	44.2%
Strongly agree	5	1.5%
Disagree	176	54%
Strongly disagree	1	0.3%
Most of friends do not believe in using condoms.		
Agree	124	38%
Strongly agree	5	1.5%
Disagree	195	59.8%
Strongly disagree	2	0.6%
How many of your friends are adolescent mothers?		
Many	31	9.5%
Some	23	7.1%
A few	164	50.3%
None	104	31.9%
Do not know	4	1.2%

### Peer influence

Table 6 shows that 231 (70.9%) of the participants disagreed that their friends believed in waiting for marriage to have sex. Furthermore, 176 (54%) disagreed that their friends believed in using condoms. Of the 326 participants, only 101 (31.9%) stated that none of their friends were adolescent mothers.

### Categorical variables included in the regression models

The following categorical variables were included in the regression models: repeat adolescent pregnancy, highest education level, the participants’ mothers’ marital status, and answering the pregnancy, HIV/AIDS and STI questions correctly. The frequency distributions of these categorical variables can be found in Table 1. Overall, only 143 (43.9%) participants answered 50% or more of the knowledge questions correctly while 183 (56.1%) answered less than 50% of the knowledge questions correctly.

### Bivariate analysis

As shown in Table 7, there was no relationship between answering the pregnancy and HIV/STI knowledge questions correctly and having a repeat adolescent pregnancy. Almost equal proportions of the participants with and without repeated pregnancies answered 50% or more questions correctly. Age was positively associated with adolescent repeat pregnancies whereby older participants had higher repeat pregnancies than their younger counterparts (*p* <  0.0001). The participants with a secondary level of education had fewer repeat pregnancies indicating that a higher education level was a protective factor against repeat pregnancies (*P* <  0.0001).

**Table 7:** Bivariate analysis (comparison of model variables for participants who had repeat pregnancies versus those who did not)

Variables	Adolescent Repeat Pregnancies		
	No *n* = 261	Yes *n* = 65	*P*-value*
Age (years)	18.0 (17.0–19.0)	19.0 (18.0–19.0)	< 0.0001
Mother’s Marital Status			0.064
Single	126 (48.3%)	21 (32.3%)	
Married	76 (29.1%)	25 (38.5%)	
Other	59 (22.6%)	19 (29.2%)	
Highest Education Level			< 0.0001
Primary	2 (0.8%)	7 (10.8%)	
Junior	6 (2.3%)	6 (9.2%)	
Secondary	253 (96.9%)	52 (80.0%)	
Answered knowledge questions correctly			1.0
50% or more correct answers	115 (44.1%)	28 (43.1%)	
Less than 50% correct answers	146 (55.9%)	37 (56.9%)	

All continuous values are reported with median and inter-quartile range, Med (IQR), while categories are reported in percentages: n (%)

*p value was obtained by Wilcoxon signed-ranked test for continuous variables and Fishers exact test for categorical variables, respectively

### Logistic regression analysis

Logistic regression was used to construct models. In each model, the outcome variable (adolescent repeat pregnancies) was regressed upon a primary ‘exposure of interest’. The exposure variables were age, education level, participants’ mothers’ marital status, and answering 50% or more of the questions correctly. The separate univariate models with each of these variables are depicted in Table 8. According to the univariate models, the participants’ age, education level and biological mothers’ marital status shared a relationship with adolescent repeat pregnancy. A multivariable model regressing adolescent repeat pregnancies (outcome of interest) on the indicator of answering 50% or more of the pregnancy, HIV/AIDS and STI questions correctly (exposure of interest) is also depicted in Table 8. This model adjusted for age, education level, and participants’ mothers’ marital status. For each model, an odds ratio for each of the variables in the model, as well as the 95% confidence intervals, were produced. In the adjusted model, the indicator for whether or not the participants answered 50% of the pregnancy, HIV/AIDS and STI knowledge questions correctly was not significant (adjusted odds ratio [OR] 1.04, 95%, CI 0.6–1.81, *p* = 0.8862).

**Table 8:** The association between answering 50% or more of the pregnancy and STI/HIV questions correctly and adolescent repeat pregnancies, adjusting for age, education, and biological mothers’ marital status

Variables	Univariate models			Multivariable logistic regression		
	OR	95% CI	*P* value	AOR	95% CI	*P* value
Age	2.69	1.85–4.47	0.0000	2.79	1.87–4.47	0.0000
Highest education level						
Primary	Ref			Ref		
Junior	0.29	0.03–1.8	0.248	0.41	0.04–3.43	0.4194
Secondary	0.06	0.01–0.25	0.0005	0.05	0.01–0.24	0.00058
Mothers’ Marital Status						
Single	Ref			Ref		
Married	1.97	1.04–3.8	0.0392	1.77	0.86–3.69	0.1207
Other	1.93	0.96–3.87	0.0626	1.97	0.01–4.3	0.08524
Answered knowledge questions correctly:						
Answered 50% or more questions correct	Ref			Ref		
Answered less than 50% of answered correctly	1.04	0.6–1.87	0.8862	0.81	0.44–1.51	0.5022

## Discussion

Knowledge plays an important role in facilitating people’s access to healthcare [16]. The aim of this study was to determine the knowledge, personal attitudes and peer influences related to pregnancy, sexual and reproductive health among adolescents who attended maternal health services in a district hospital in Ugu, KwaZulu-Natal, South Africa. Although 43.9% of participants scored > 50% for the knowledge test on pregnancy, HIV/AIDS and STIs, there are serious gaps in their knowledge of danger signs of pregnancy, anaemia, alcohol and tobacco use during pregnancy and sexually transmitted infections.

In our study, 60.4% of the participants was able to identify vaginal bleeding as a sign of preterm labour. In reviewing the literature, this is low when compared to 86.0 and 82.2% who were able to identify vaginal bleeding as a danger sign during pregnancy in studies conducted in Malaysia and Tanzania respectively [16, 17]. The current study found that only 23.3% of the participants was able to identify blurred or spotted vision as an abnormal sign during pregnancy in comparison to 47.8 and 39.4% in studies conducted in Malaysia and Ethiopia respectively [16, 23]. Prior studies have noted the importance of pregnant women having adequate pregnancy related knowledge to identify the appropriate steps to decrease the risk of preterm labour. In this study, 44.2% of the participants was able to identify the appropriate steps to decrease the risk of preterm labour. This result is in concordance with the level of pregnancy related knowledge among pregnant women attending an antenatal clinic in Pune, Maharashstra [24]. The ability of pregnant women to identify the signs of preterm labour is critical if they are to counteract this emergency.

Anaemia is associated with adverse events in pregnancy and is common among pregnant adolescents. [25]. The participants in the current study demonstrated poor knowledge of the signs and symptoms of anaemia. More than half (63.2%) was not aware that iron deficiency can lead to anaemia. This is in contrast to the findings in Brosankro, Ghana, where only 3% of the participants in a study, was unaware of the link between iron deficiency and anaemia [26]. In the current study, 59.5% of the participants could identify only one out of thirteen signs and symptoms of anaemia. Less than half of the participants in this study identified pale lips (31.3%) and pale finger nails (29.8%) as symptoms of anaemia. This is low in comparison to 87% of participants who was aware of this fact in a study in Dahalik, Baghdad [27]. In the current study, 43.3% of the participants identified preterm delivery and low birth weight as complications of anaemia, in comparison to 25.0 and 11.0% who respectively correctly identified preterm labour and low birth weight as complications of anaemia in a Ghanaian study [26]. Moreover, knowledge of the benefits of folic acid supplementation and neural tube defects is important, yet this study found that only 35.6% of the participants knew of the benefits of folic acid for brain and spine development. This was slightly better than the findings of a study conducted in Pakistan, in which only 18.4% of the participants knew of the importance of folic acid for the unborn foetus [28]. A study conducted in Sudan found that only 8.9% of 1000 pregnant women knew that folic acid prevented birth defects [25].

Alcohol and tobacco use during pregnancy is detrimental to the health of the unborn child [29]. Persistent tobacco inhalation can cause preterm labour, low birth weight, intra-uterine growth retardation, and spontaneous abortion [30]. It is also related to foetal alcohol syndrome [31]. The prevalence of foetal alcohol spectrum disorder (FASD) in South Africa ranges from 29 to 290 per 1000 live births [32]. Many research studies have focused on the population’s knowledge of the harmful effects of smoking and alcohol use among adult women of reproductive age. However, research data of knowledge of this threat among pregnant and parenting adolescent women are limited. This is a matter of grave concern, as this study found that only 19.6% of the participants knew that alcohol consumption results in low birth weight. A national survey among Australian women aged 18 to 45 years revealed that only 28.5% knew that drinking alcohol during pregnancy could result in low birth weight [31]. Furthermore, the participants’ knowledge of the negative effects of tobacco during pregnancy on the unborn child was poor, as only 22.4, 16.6 and 6.7% identified that smoking tobacco during pregnancy can result in low birth weight, spontaneous abortion and preterm labour respectively. These findings are low compared to those of a US study among pregnant adolescents and adolescent mothers that found that 79.6, 85.3 and 89.0% were able to correctly identify that smoking during pregnancy results in low birth weight, spontaneous abortion and preterm labour respectively [33].

In our study, 42.9% of the participants knew that pesticides should be avoided, compared to 96.0% who knew this in a survey conducted in Northern Thailand [34]. However, regardless of their high knowledge of the harmful effects of pesticides to the unborn child, 37.0% of participants in Thailand admitted that they still used pesticides in their homes during pregnancy [34]. The knowledge of the harmful effects of pesticides to the unborn child is of public health importance due to neurodevelopmental toxicity [34]. Cats harbour the protozoan parasite *Toxoplasma Gandii* in their intestinal tract [35] that causes Toxoplasmosis. Toxoplasmosis infection during pregnancy can cause spontaneous abortion, preterm labour, cerebrospinal fluid abnormalities, retinochoroiditis scarring, and intra-uterine growth retardation [35]. Respectively, only 41.1 and 37.0% of the participants in this study agreed that playing with cats and cleaning a cat’s litter box should be avoided. This rate is low compared to the 77.9% of participants who correctly indicated that a pregnant woman should avoid cleaning a cat’s litter box in a study conducted in the Netherlands [36]. The contrast in these findings could be due to a higher literacy rate and access to antenatal education in the Netherlands.

The World Health Organization recommends exclusive breastfeeding for the first 6 months as a global strategy for infant and young child feeding [37]. Breastfeeding has numerous health benefits for the mother and child. The benefits for the child include reduction of dental cavities, improved immunity, and protection against the risk of diabetes and obesity later in life [38]. Most studies to date have investigated knowledge of breast feeding practices rather than knowledge of the health benefits of breastfeeding. In this study, 71.8% correctly identified that the recommended period for exclusive breast feeding is 6 months. Similarly, 70.13% of participants in a study conducted in Croatia identified that exclusive breast feeding is recommended for 6 months [39]. Most of the participants (78.8%) in this study agreed that breastfeeding improves the immunity of babies. Although somewhat lower, this finding was high enough to support the 92.9% of a Croatian study [39].

STIs increase the risk of HIV infection, pelvic inflammatory diseases, life threatening pregnancies, and infertility [40]. Adolescents have a higher risk of STIs than adults [41]. In this study, the participants were unable to identify common STIs as only 24.5, 15.0 and 15.6% were able to identify Gonorrhoea, Syphilis, and Genital Herpes as STIs respectively. These findings are consistent with those of a study conducted in Nigeria where 23.0% identified Gonorrhoea, 2.8% identified Syphilis, and 6.5% identified Genital Herpes as STIs [42]. However, the ability to identify signs and symptoms of STIs was better as most participants were able to correctly identify genital sores (57.1%, *n* = 186), burning urination (54.9%, *n* = 179), discharge from the vagina (65.6%, *n* = 214), and discharge from the penis (59.8%, *n* = 195) as symptoms of STIs. These response rates were far better than those found by Sharma and Sherkane [40], in whose study only 22.8, 41.7 and 13.9% identified genital sores, genital discharge and burning urination respectively as signs and symptoms of STIs.

The prevalence of HIV amongst women of reproductive age is high in South Africa [43]. In this study, 70.2% of the participants was aware that a pregnant woman infected with HIV can transmit this disease to her unborn child, which was high in comparison to the 51.6% in a study conducted in India [44]. Most participants (92.6%) in the current study confirmed that condom use can prevent HIV and STIs, in comparison to 55.6% who had this knowledge during a study conducted in Nepal [45]. With regards to HIV prevention, 7.7% thought that birth control pills would prevent HIV infection. This is low compared to the 41% in a study conducted in South Delhi, India, who incorrectly believed that birth control pills would prevent HIV infection [46].

A recent study by Amod et al. explored adolescent mothers’ perceptions of the Life Orientation curriculum in Gauteng, South Africa [47]. The participants felt that although sexual health was included in Life Orientation, their teachers were not comfortable to teach them about sex education. Furthermore, the participants reported that learners would laugh during the lesson and this annoyed the teacher. Under these circumstances, the lessons on sexual health would be dismissed. Similarly, a study on learners’ experiences of Life Orientation in the North-West Province reported that 33.3% of learners mentioned that sex education was covered in Life Orientation [48]. In this study, 87.7% mentioned that adolescent pregnancy was covered in Life Orientation, while 77.9% mentioned that HIV/AIDS was covered in Life Orientation. Contrary to the findings of our study, 33.8% of the North-West study stated that HIV/AIDS was covered and 22% stated that adolescent pregnancy was covered in Life Orientation.

On face value, the Life Orientation programme appears encouraging, but the practical implementation of this programme has been plagued by many problems, and thus the limited research that has been done on its efficacy in terms of SRH education should be augmented as a matter of urgency.

Personal attitudes towards sexuality and reproductive health is often under-researched. In this study, only 26.7% agreed that abstaining from sex is difficult during adolescent years. This suggests that most participants had a positive attitude towards abstinence. Similarly, a study by Masters et al. [49] found that more females than males reported positive attitudes towards abstinence (means 3.6 vs 4.0 on the five-point Likert scale, where a score of 3 was neutral). In the current study, only 16.3% believed that condoms reduce sexual pleasure during sexual intercourse. During a literature review regarding decision making and condom use among South African women, Mash et al. [50] found that men were more likely to believe that condoms reduce sexual pleasure than women. In a patriarchal society, South African women are disempowered and more likely to please their partners by agreeing not to use condoms during sexual intercourse [50]. Thus gender based power inequalities were clearly voiced when 66.3% of the participants in this study agreed that it is females who are responsible for protection during sexual intercourse. Furthermore, 23.9% indicated that they would be too embarrassed to buy or find condoms, which suggests that they were not in a situation to negotiate protective sexual intercourse.

Peer influence is related to sexual risk behaviour and adolescent contraceptive behaviour is also influenced by peer norms. Govender et al. [51] conducted a scoping review and found that adolescents who associated with friends who were adolescent parents were more likely to become adolescent parents themselves. In the current study, 66.8% of the participants had friends who were adolescent mothers which, based on the former study, may suggest that they might be at risk of engaging in unprotected sexual behaviour as well.

The participants limited knowledge of preterm labour, anaemia, alcohol and tobacco use, and sexually transmitted infections may hamper the Safe Motherhood Initiative. The misconceptions harboured by these adolescents about oral contraception and protection against HIV and STIs also suggest the threat of unsafe sexual practices among young people in the Ugu community. Health education on SRH is an integral part of the Safe Motherhood Initiative that is aimed at educating societies about safe sexual conduct.

The study has several limitations. Firstly, our study was conducted in only one district in KwaZulu-Natal and therefore the findings may only be generalised to the Ugu and similar districts. Moreover, the study did not investigate the participants’ sources of pregnancy or information regarding their sexual and reproductive health. Recall bias was likely to have occurred when participants are asked about their past exposures. The use of convenience sampling could have also lead to under-representation or over-representation of particular groups within a sample. Although our inclusion criteria stipulated that adolescent girls under the age of 18 years had to obtain parental/legal guardian permission prior to consenting to participate in this study, we report that there were no participants under 18 years who were excluded in the study because parents and legal guardians were available to provide written permission.

## Conclusion

The adolescent participants’ knowledge of pregnancy, sexual and reproductive health was deficient in many respects. Our research has shown that, regardless of repeat pregnancies, adolescents were not necessarily better informed about pregnancy, sexual and reproductive health. Thus we conclude that social determinants, modes and platforms regarding the delivery of adolescent sexual and reproductive health education have become more important than ever before. Moving forward, an innovative mode to the delivery of sexual and reproductive health education includes the emerging digital platform [1, 52]. The digital platform encompasses social media, multimedia and mobile phones which is growing popular among young people. Previous studies in Sub-Saharan Africa have demonstrated the feasibility of using mobile phones to deliver SRH information [53, 54].

Schools can play a role in reducing high risk sexual behaviour, transforming the future and improving the well-being of all adolescents. The role of the education sector is therefore crucial as schools can offer skills based SRH education. Schools are also an ideal social environment that can target the individual, families and societies [15]. The role of the healthcare sector is equally important. Health facilities need to be adolescent or youth friendly with convenient service hours. Healthcare providers also need to provide non-judgemental adolescent SRH education and services. Universal access to SRH as echoed in SDG 3 can only be achieved through intersectoral collaboration. Knowledge through education is likely to ensure that adolescent women are better informed to make appropriate decisions about their health during pregnancy and childbirth.

## Additional file

Additional file 1: Adolescent pregnancy, sexual and reproductive health questionnaire. (DOCX 69 kb)

## Data Availability

The data used to elicit the findings of this study are available from the corresponding author upon reasonable request.

## Abbreviations

AIDS: Acquired Immunodeficiency Syndrome
HIV: Human Immunodeficiency Virus
MMSE: Modified mini mental state exam
SRH: Sexual and Reproductive Health
STI: Sexually transmitted disease
