# Supplementary material for: Knowledge, attitudes and peer influences related to pregnancy, sexual and reproductive health among adolescents using maternal health services in Ugu, KwaZulu-Natal, South Africa
Source: BMC Public Health. 2019 Jul 11;19:928. doi: 10.1186/s12889-019-7242-y (PMC6621947; doi:10.1186/s12889-019-7242-y)
Supplement: Supplementary file 1 — Adolescent pregnancy, sexual and reproductive health questionnaire. (DOCX 69 kb) [file 12889_2019_7242_MOESM1_ESM.docx]

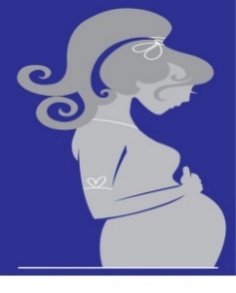


**Additional file 1: Adolescent pregnancy, sexual and reproductive health questionnaire**

**Socio-Demographic Characteristics**

**The questions in this section are about your and your family’s characteristics as well as your socio-economic status. Please indicate your answer with an X next to the option(s) of your choice, or fill in the blank space**.

1. How old are you?_______ years
2. What is your religion?

| Christianity | Judaism |
| --- | --- |
| Islam | Other (please specify): |
| Hinduism |  |

1. What is your race group?

| African | White | Indian | Coloured | Other: |
| --- | --- | --- | --- | --- |

1. What is your home language?

| English | Zulu | Xhosa | Afrikaans | Other: |
| --- | --- | --- | --- | --- |

1. What is your present marital status?

| Single | Married | Divorced | Separated | Widowed |
| --- | --- | --- | --- | --- |

***Clinical information***

1. Obstetric History:

| Number of pregnancies |  |
| --- | --- |
| Number of deliveries |  |
| Number of live births |  |
| Number of stillbirths |  |
| Number of abortions |  |

1. Current medical problems:

|  | Yes | No | Don’t know |
| --- | --- | --- | --- |
| High blood pressure |  |  |  |
| Diabetes |  |  |  |
| Heart disease |  |  |  |
| Varicose veins |  |  |  |
| Anaemia |  |  |  |
| Seizures |  |  |  |
| Asthma |  |  |  |
| Tuberculosis |  |  |  |
| Sexually transmitted infection(s) |  |  |  |
| HIV |  |  |  |
| Other medical problems: | | | |

***Your living arrangements***

1. Which of the following is/are applicable to your living situation? (Indicate all those that apply.)

|  | I live alone |
| --- | --- |
|  | I live with my parents |
|  | I live with my father only |
|  | I live with my mother only |
|  | I live with my mother and stepfather |
|  | I live with my father and stepmother |
|  | I live with my grandparents/a grandparent |
|  | I live with a sibling/siblings |
|  | I live with other relatives |
|  | I live with my husband |
|  | I live with my partner |
|  | I live with my partner and his family |
|  | I live with friends |
|  | Other (specify): |

***Family characteristics***

1. Who has been responsible for your upbringing since childhood?

| Both  parents | Mother & stepfather/s | Father & stepmother/s | Single father | Single mother | Grandparent/s | Siblings | Other relatives | other: |
| --- | --- | --- | --- | --- | --- | --- | --- | --- |

1. What is the current marital status of your biological or adoptive mother?

| Single  (never married) | Married | Divorced | Divorced and remarried | Married but  Separated | Deceased |
| --- | --- | --- | --- | --- | --- |

1. What is the current marital status of your biological or adoptive father?

| Single | Married | Remarried | Divorced | Separated | Deceased |
| --- | --- | --- | --- | --- | --- |

1. What is the highest level of education completed by your mother?

| None | Primary school  (grades 1-5) | Junior phase  (grades 6-8) | Secondary  (grades 9-12) | College or technical school certificate | College diploma | University degree |
| --- | --- | --- | --- | --- | --- | --- |

1. What is the highest level of education completed by your father?

| None | Primary school  (grades 1-5) | Junior phase  (grades 6-8) | Secondary  (grades 9-12) | College or technical school certificate | College diploma | University degree |
| --- | --- | --- | --- | --- | --- | --- |

1. How many children does your biological mother have (including yourself)? ___________
2. How old was your mother when she had her first child? _____years /____don’t know.
3. Did any of your siblings become parents during their adolescent phase (13 – 19 years of age)?

| Yes | No | Don’t know |
| --- | --- | --- |

1. Did you experience violence in one or more of the following forms while growing up? Please indicate which is applicable to your experiences.

| Form of violence | Yes | No | I wish not to answer |
| --- | --- | --- | --- |
| Physical |  |  |  |
| Sexual |  |  |  |
| Verbal |  |  |  |

***Socio-economic status***

1. Please indicate all the people that live in your household (i.e., people who eat and sleep at home at least 4 days a week). Refer to the rating indicators below for your responses.

| **Relationship of household members to you** | **Age** | **Gender**  **(Male/Female)** | **Employment status**  **(Yes or No)** | **Level of education** |
| --- | --- | --- | --- | --- |
|  |  |  |  |  |
|  |  |  |  |  |
|  |  |  |  |  |
|  |  |  |  |  |
|  |  |  |  |  |
|  |  |  |  |  |
|  |  |  |  |  |
|  |  |  |  |  |
|  |  |  |  |  |
|  |  |  |  |  |
|  |  |  |  |  |
|  |  |  |  |  |
| 1=father  2=mother  3=sibling (brother/sister)  4=grandmother  5=grandfather  6=aunt  7=uncle  8=cousin  9=friend  10=partner | If unsure of age:  1=adult  2=child  3=don’t know | M = male  F = female | 1=employed  2=self-employed  3=part –time employed  4=unemployed  5=pensioner  6=child  7=school going learner  8=student at tertiary  institution  9=don’t know  10=dependent on a grant | 1=preschool  2=primary school  3=secondary  school  4=tertiary  education  5=don’t know |

1. Who is the current head of the household?

| Father |  | Uncle |  |
| --- | --- | --- | --- |
| Mother |  | Cousin |  |
| A sibling |  | Friend |  |
| Grandmother |  | Partner |  |
| Grandfather |  | My husband |  |
| Aunt |  | Other: |  |

1. How many people contribute to the total income of the household?

| 1 | 2 | 3-4 | 5-6 | More than 6 |
| --- | --- | --- | --- | --- |

1. Please indicate the type of dwelling you live in:

| Formal house | Flat/apartment | Informal dwelling | Wendy house | Homeless | Other : |
| --- | --- | --- | --- | --- | --- |

1. What materials have been used to construct the walls of your dwelling?

| Wood and mud | Bricks and plaster |
| --- | --- |
| Stones and mud | Plastic |
| Stones and cement | Cardboard |
| Cement and blocks | Corrugated iron |
| Wooden poles | Don’t know |

1. What materials have been used in the construction of the roof of your dwelling?

| Thatch | Plastic |
| --- | --- |
| Tin | Cardboard |
| Poles | No roof |
| Tiles | Don’t know |

1. What is the number of bedrooms in the house? ______
2. What is the number of people per bedroom?

| 0-2 | 3-4 | more than 4 |
| --- | --- | --- |

1. Where do you get drinking water from most of the time?

| Own tap | River/Dam |
| --- | --- |
| Communal tap | Borehole/Well |

1. What type of toilet does the dwelling have?

| Flush toilet indoors | Pit latrine |
| --- | --- |
| Flush toilet outdoors | Bucket system |
| No toilet | Other: |

1. What fuel is used for cooking most of the time?

| Wood/coal | Electricity |
| --- | --- |
| Open fire | Gas |
|  | Paraffin |

1. Which of the following items are available (in a working condition) in your house?

| Radio | Refrigerator |
| --- | --- |
| TV | Electric stove |
| Landline telephone | Paraffin stove |
| Cellphone | Microwave oven |
| Car | Computer |
| Coal stove | Wood stove |

***Schooling and Employment***

1. Which highest level of schooling did you complete?

| None | Junior (grades 6-8) |
| --- | --- |
| Primary (grades 1-5) | Secondary (grades 9-12) |

1. Are you currently employed? YES ________NO _______ (if the answer is NO, leave out Question 32)
2. Is your current employment fulltime or part-time?

|  | Fulltime |
| --- | --- |
|  | Part-time |

1. Are you currently attending school? YES _______NO _______ (if the answer is NO, leave out question 34).
2. How would you rate your school attendance since you became pregnant?

| I go to school almost every day | I miss school 3 days a week |
| --- | --- |
| I miss school 2 days a week | Doesn’t apply ( I am not in school) |

***Social and Financial Support***

1. Who knows about your pregnancy and how would you describe their emotional support to you during the pregnancy? (You may list as many people as you wish.)

| Relationship of the person to you who knows about your pregnancy | Not supportive | Moderately supportive | Very supportive |
| --- | --- | --- | --- |
|  |  |  |  |
|  |  |  |  |
|  |  |  |  |
|  |  |  |  |
|  |  |  |  |
|  |  |  |  |
|  |  |  |  |
|  |  |  |  |
|  |  |  |  |
|  |  |  |  |
|  |  |  |  |
| 1=mother  2=father  3=brother  4=sister  5=grandmother  6=grandfather  7=cousin  8=aunt  9=uncle  10=partner/boyfriend  11=best friend  12=teacher | | | |

1. What are all your current sources of financial support?

| Your job | Grandparents |
| --- | --- |
| Savings | Baby’s father |
| Loans | Friends |
| Social Services | Other relatives (please specify) |
| My parents |  |
| Siblings (brother/sister) |  |
| Church | Parents of baby’s father |

1. Which of the following person/s do you think will provide you and your child/children with support (e.g., advice, emotional support, financial support and baby care)?

| No one | Grandfather |
| --- | --- |
| Father | Aunt |
| Mother | Uncle |
| Sibling | Cousin |
| Grandmother | Partner |
| Friend | Other (please specify) |

***The Baby’s Father***

1. This question applies only to adolescents with repeat pregnancies:

Is the father of your previous pregnancy responsible for your recent pregnancy?

| Yes | No | Don’t know |
| --- | --- | --- |

1. What is the age of your baby’s father? ________ years
2. What is the employment status of your baby’s father?

| Employed fulltime | Student at tertiary institution |
| --- | --- |
| Employed part-time | Other |
| Unemployed |  |
| Learner at school |  |

1. What is your relationship with your baby’s father?

| No relationship | Married and not living together (separated) |
| --- | --- |
| Married and living together | Not living together and not married |
| Living together and not married | Widowed |
| Divorced |  |

1. What is the extent of your contact with your baby’s father?

| See him every day | Never see him |
| --- | --- |
| See him once a week | Not living together and not married |
| See him 2 or 3 times a week | Widowed |
| See him once or twice a month | Not applicable |
| See him every day | Other (please specify) |
|  |  |
|  |  |

1. Has the father of your baby been violent towards you?

| Yes | No | I refuse to answer this question |
| --- | --- | --- |

1. How much support do you think you will receive from the baby’s father in taking care of the baby?

| None | Reasonable support |
| --- | --- |
| Little support | A lot of support |

***Use of Contraception***

1. Please indicate which of the following are contraception methods:

| The pill | Diaphragm |
| --- | --- |
| I.U.D. | Withdrawal |
| Male Condom | Rhythm |
| Female Condom | Injection |
| Foam Spermicide | Implanon |
| All the above mentioned |  |

1. Were you using a contraceptive method or methods at the time you became pregnant? YES _____ NO _____
2. If yes, what type of contraception method/s have you used in the last 12 months?

| The pill | Rhythm |
| --- | --- |
| I.U.D. | Injection |
| Male Condom | Implanon |
| Female Condom | Diaphragm |
| Foam Spermicide | Withdrawal |
| Other (please specify): |  |
|  |  |

1. Where do you commonly obtain contraceptives?

| Local clinic | Other (please specify) |  |
| --- | --- | --- |
| Local hospital | I have never used contraception so this does not apply to me |  |
| Family doctor |  |  |

1. How easy is it for you to get hold of contraceptives?

| Difficult | Fair | Good | Excellent | I do not  know |
| --- | --- | --- | --- | --- |

1. How would you rate your knowledge about contraception?

| Poor | Fair | Good | Excellent |
| --- | --- | --- | --- |

1. In your opinion, do you feel that contraception is:

| Your responsibility | Your partner’s responsibility | Shared responsibility |
| --- | --- | --- |

***Pregnancy-related knowledge***

Questions have been adopted and adapted from: Godin, K; Blue, S; Bourdages, N; Edwards, S; Horan, M; MacDougall, R; Mill, C; Procter, TD; Schieck, A; Alton, GD. (2014). Assessing Public Health Prenatal Education Knowledge: Findings from the LDCP Healthy Pregnancies Project. Woodstock, Ontario, Canada.

The following questions relate to your knowledge of pregnancy. Please mark an X next to your answer/s. You may indicate more than one option unless ONLY ONE answer is required.

1. A pregnant woman must consult a clinician (doctor or midwife/nurse) as soon as possible in her pregnancy.

| True | False | Do not know |
| --- | --- | --- |

1. A pregnant woman must consult her clinician (doctor or midwife/nurse) in the event she feels unwell, has a fever, and/or experiences an unusual change.

| True | False | Do not know |
| --- | --- | --- |

1. Which of the following are signs of preterm (too early) labour?

| Persistent cramps and abdominal (stomach) pain | A feeling like the baby is pushing down towards the cervix |
| --- | --- |
| The release of fluid or blood from the vagina | Swollen ankles |
| Regular or frequent contractions or changes in the strength or number of contractions. | Don’t know |

1. What are the most important things that a pregnant woman should do if she is experiencing all the signs and symptoms of preterm labour?

| Contact the doctor or midwife/nurse. | She should have a meal. |
| --- | --- |
| Go to the hospital immediately. | She should take some pain medication. |
| Take a nap and rest. | Do not know. |

1. Select ONLY ONE answer from the options given:

To decrease the risk of preterm labour, a pregnant woman can…

| attend antenatal care as soon as she knows she is pregnant. | eat healthy, nutritious food. |
| --- | --- |
| reduce stress. | All of the above. |
| follow all the health care advice given by the doctor or midwife. | Do not know. |

1. Aches and discomfort in the hips and lower back are normal during pregnancy.

| True | False | Do not know |
| --- | --- | --- |

1. Nausea is normal during pregnancy.

| True | False | Do not know |
| --- | --- | --- |

1. Fatigue (tiredness) is normal during pregnancy.

| True | False | Do not know |
| --- | --- | --- |

1. Abnormal vaginal discharge with an odour or blood is normal during pregnancy.

| True | False | Do not know |
| --- | --- | --- |

1. Spotted vision is normal during pregnancy.

| True | False | Do not know |
| --- | --- | --- |

1. Swelling of the face and hands is normal during pregnancy.

| True | False | Do not know |
| --- | --- | --- |

1. Select ONLY ONE of the options given:

Folic acid is taken by pregnant women because it…

| prevents nausea. | helps with the baby’s lung development |
| --- | --- |
| is essential for the baby’s brain and spine development. | helps to reduce lower back pain. |
| prevents sexually transmitted infections. | Do not know |

1. Iron deficiency leads to anaemia.

| True | False | Do not know |
| --- | --- | --- |

1. Which of the following are signs and symptoms of anaemia?

| Rapid heartbeat. | Pale fingernails |
| --- | --- |
| Shortness of breath | Pale skin |
| Headaches | Pale lips |
| Dizziness | Paleness of the underside of the eyelids |
| Irritability | Pale skin |
| Trouble concentrating | Numb or cold feeling in hands and feet |
| Cravings to eat non-food items (pica) such as sand or paper | Do not know |
| Chest pain |  |

1. Anaemia during pregnancy can result in preterm delivery and low birth weight.

| True | False | Do not know |
| --- | --- | --- |

1. Every pregnant woman should avoid all physical activity/exercises during pregnancy.

| True | False | Do not know |
| --- | --- | --- |

1. What quantity of alcohol may a pregnant woman consume? Select ONLY ONE answer from the options given:

| One alcoholic drink a day | No quantity of alcohol is safe during pregnancy. |
| --- | --- |
| 2-3 alcoholic drinks per week | Do not know |
| One alcoholic drink per month |  |

1. Which of the following may be the result of the consumption of alcohol during pregnancy?

| Low birth weight | Mental retardation in the unborn baby |
| --- | --- |
| Heart defects in the baby | Do not know |
| Damage to the unborn baby’s liver |  |

1. Which of the following may be the result of smoking tobacco during pregnancy?

| Low birth weight | Deformities to the baby in the uterus |
| --- | --- |
| Spontaneous abortion (miscarriage) | Reduction in lower back pain |
| Preterm labour | Do not know |

1. Which of the following should be avoided by a pregnant woman?

| Washing clothes | Cleaning of a cat’s litter box |
| --- | --- |
| Using pesticides | X-rays |
| Playing with cats | Do not know |

1. Which length of time is recommended for exclusive breastfeeding? Select ONLY ONE answer from the options given:

| 6 weeks | 6 months |
| --- | --- |
| 2 months | 8 months |
| 4 months | Do not know |
|  |  |

1. Breastfeeding improves the immunity of the baby and therefore protects the baby from allergies and asthma.

| True | False | Do not know |
| --- | --- | --- |

1. A breast-fed baby has a lower risk of obesity and diabetes.

| True | False | Do not know |
| --- | --- | --- |

1. Breastfeeding is more time consuming than bottle feeding.

| True | False | Do not know |
| --- | --- | --- |

1. Breast-fed babies have fewer dental cavities (i.e., problems with tooth decay).

| True | False | Do not know |
| --- | --- | --- |

1. Drugs and alcohol can be passed through breast milk to the baby.

| True | False | Do not know |
| --- | --- | --- |

***Knowledge of HIV/AIDS/STIs:***

1. Which of the following are known sexually transmitted infections (STIs)?

| Gonorrhoea | Genital herpes |
| --- | --- |
| Syphllis | Do not know |
| Chlamydia |  |

1. Which of the following signs and symptoms are associated with STIs?

| Genital sores | Discharge from penis |
| --- | --- |
| Burning sensation during urination | Do not know |
| Discharge from the vagina |  |

1. HIV is known as the Human Immunodeficiency Virus.

| True | False | Do not know |
| --- | --- | --- |

1. Through which of the following can HIV be transmitted?

| Oral sex | Anal sex |
| --- | --- |
| Vaginal sex | Do not know |

1. HIV infects and damages the CD4 helper cells.

| True | False | Do not know |
| --- | --- | --- |

1. HIV develops into AIDS

| True | False | Do not know |
| --- | --- | --- |

1. Which of the following signs and symptoms are associated with AIDS?

| Rapid weight loss | White sores in the mouth and throat |
| --- | --- |
| Extreme weakness | Sores in the genital and anal regions |
| Chronic diarrhoea | Do not know |

1. HIV is present in semen, blood, vaginal secretions and breast milk

| True | False | Do not know |
| --- | --- | --- |

1. Condoms can help prevent HIV/AIDS and other STIs

| True | False | Do not know |
| --- | --- | --- |

1. Birth control pills can prevent HIV/AIDS and other STIs.

| True | False | Do not know |
| --- | --- | --- |

1. All sexually active women must have an annual pap smear.

| True | False | Do not know |
| --- | --- | --- |

1. How can an individual avoid getting HIV/STI?

| Abstain from sexual intercourse. | Use condoms every time you have sexual intercourse. |
| --- | --- |
| Be faithful to one partner. | Refuse to share needles. |
| Do not know |  |

1. If the signs and symptoms of an STI disappear without medical treatment, then the infection is cured.

| True | False | Do not know |
| --- | --- | --- |

1. A person can become infected with an STI or HIV by…

| abstaining from sexual intercourse | refusing to share needles |
| --- | --- |
| being faithful to one partner | None of the above |
| using a condom every time you have sexual intercourse | Do not know |

1. A pregnant woman who is infected with HIV/STIs can transmit these diseases to her unborn baby.

| True | False | Do not know |
| --- | --- | --- |

***Sexual risk behaviour***

1. In the last 12 months, how often did you have unprotected sex with more than one sexual partner?

| Never | Rarely | Sometimes | Often | Always |
| --- | --- | --- | --- | --- |

***Personal attitudes towards sexuality and reproductive health***

1. Sex before marriage is acceptable.

| Agree | Strongly agree | Disagree | Strongly disagree |
| --- | --- | --- | --- |

1. For a woman, having multiple sex partners is an indication of her attractiveness.

| Agree | Strongly agree | Disagree | Strongly disagree |
| --- | --- | --- | --- |

1. It is important for a man to have multiple sex partners to prove his manhood.

| Agree | Strongly agree | Disagree | Strongly disagree |
| --- | --- | --- | --- |

1. A female who remains a virgin during her adolescence is old fashioned.

| Agree | Strongly agree | Disagree | Strongly disagree |
| --- | --- | --- | --- |

1. Having a baby at an early age is a sign of maturity.

| Agree | Strongly agree | Disagree | Strongly disagree |
| --- | --- | --- | --- |

1. Abstaining from sex is difficult during adolescence.

| Agree | Strongly agree | Disagree | Strongly disagree |
| --- | --- | --- | --- |

1. Using a condom during sexual intercourse reduces sexual pleasure.

| Agree | Strongly agree | Disagree | Strongly disagree |
| --- | --- | --- | --- |

1. The use of contraceptives causes sterility in women.

| Agree | Strongly agree | Disagree | Strongly disagree |
| --- | --- | --- | --- |

1. Family planning services can help prevent an unwanted pregnancy.

| Agree | Strongly agree | Disagree | Strongly disagree |
| --- | --- | --- | --- |

1. I would be too embarrassed to buy or find condoms.

| Agree | Strongly agree | Disagree | Strongly disagree |
| --- | --- | --- | --- |

1. My partner will reject me if I ask him to use a condom.

| Agree | Strongly agree | Disagree | Strongly disagree |
| --- | --- | --- | --- |

1. Females are responsible for protection during sexual intercourse.

| Agree | Strongly agree | Disagree | Strongly disagree |
| --- | --- | --- | --- |

***Peer influence***

1. Most of my friends do believe in waiting for marriage to have sex.

| Agree | Strongly agree | Disagree | Strongly disagree |
| --- | --- | --- | --- |

1. Most of my friends do not believe in using contraception.

| Agree | Strongly agree | Disagree | Strongly disagree |
| --- | --- | --- | --- |

1. Most of my friends do not believe in using condoms.

| Agree | Strongly agree | Disagree | Strongly disagree |
| --- | --- | --- | --- |

1. How many of your friends are adolescent mothers?

| Many | Some | A few | None | Do not know |
| --- | --- | --- | --- | --- |

***Edinburgh Postnatal Depression Scale***

Source: Cox JL, Holden JM & Sargovsky R, 1987. Detection of postnatal depression: Development of a 10-item Edinburgh Scale. *British Journal of Psychiatry*, *150*:782-786.

***Ask the patient what she has felt in the past seven days, not just how she feels today.***

*Each question is scored on a scale of 0, 1, 2, and 3.*

*If the score is above 12, the patient is at risk of depression. Liaise with an operational manager in the division about management of the patient.*

*IF the score is higher than zero for question 10, immediately refer the patient to a mental health practitioner.*

1. In the past 7 days I have been able to laugh and see the funny side of things.

| As much as I always could = 0 | Not quite so much =1 | Definitely not so much =2 | Not at all =3 |
| --- | --- | --- | --- |

1. I have looked forward with enjoyment to things.

| As much as I ever did =0 | Rather less than I used to =1 | Definitely less than I used to =2 | Hardly at all =3 |
| --- | --- | --- | --- |

1. I have blamed myself unnecessarily when things went wrong.

| Yes, most of the time =3 | Yes, some of the time =2 | Not very often=1 | No, never =0 |
| --- | --- | --- | --- |

1. I have been anxious and worried for no good reason.

| No, not at all = 0 | Hardly ever=1 | Yes, sometimes =2 | Yes, very often =3 |
| --- | --- | --- | --- |

1. I have felt scared or panicky for no good reason.

| Yes, quite a lot=3 | Yes, sometimes =2 | No, not much =1 | No, not at all =0 |
| --- | --- | --- | --- |

1. Things have been getting on top of me.

| Yes, most of the time I haven’t been able to cope=3 | Yes, sometimes I haven’t been able to cope as well as usual =2 | No, most of the time I have coped quite well =1 | No, I have been coping as well as ever =0 |
| --- | --- | --- | --- |

1. I have been so unhappy that I have difficulty sleeping.

| Yes, most of the time =3 | Yes, sometimes =2 | Not very often =1 | No, not at all=0 |
| --- | --- | --- | --- |

1. I have felt sad or miserable.

| Yes, most of the time =3 | Yes, quite often =2 | Not very often =1 | No, not at all=0 |
| --- | --- | --- | --- |

1. I have been so unhappy that I have been crying.

| Yes, most of the time =3 | Yes, quite often =2 | Only occasionally =1 | No, never =0 |
| --- | --- | --- | --- |

1. The thought of harming myself has occurred to me.

| Yes, quite often =3 | Sometimes =2 | Hardly ever =1 | Never=0 |
| --- | --- | --- | --- |

***Health practices during pregnancy***

The questions have been adopted and adapted from:

Lindgren, K. (2005). Testing the health practices in pregnancy in questionnaire-II. *Journal of Obstetric Gynecologic and Neonatal Nursing, 32*(3):313-321.

1. Since knowing about your pregnancy, how often have you exercised at least 3 times per week?

| Never | Rarely | Sometimes | Often | Always |
| --- | --- | --- | --- | --- |

1. Since knowing about your pregnancy, how often have you used dagga?

| Never | Rarely | Sometimes | Often | Every day |
| --- | --- | --- | --- | --- |

1. Since knowing about your pregnancy, how often have you used illegal drugs (other than dagga) such as cocaine, “sugars”, whoonga, etc.?

| Never | Rarely | Sometimes | Often | Every day |
| --- | --- | --- | --- | --- |

1. How often have you spoken to your doctor or midwife about concerns relating to your pregnancy?

| Never | Rarely | Sometimes | Often | Every week |
| --- | --- | --- | --- | --- |

1. How often have you asked your doctor or midwife questions about your pregnancy that you don’t understand?

| Never | Rarely | Sometimes | Often | Every week |
| --- | --- | --- | --- | --- |

1. How often have you discussed the use of medication and supplements with your doctor or midwife to ensure that these are safe for you and the baby?

| Never | Rarely | Sometimes | Often | Every week |
| --- | --- | --- | --- | --- |

1. Since knowing about your pregnancy, have you used any traditional herbs/medicinal plants that are not recommended?

| Never | Rarely | Sometimes | Often | Every day |
| --- | --- | --- | --- | --- |

1. Since knowing about your pregnancy, have you limited or avoided use of dangerous substances (such as cigarette smoke, insecticides, and pesticides)?

| Never | Rarely | Sometimes | Often | Always |
| --- | --- | --- | --- | --- |

1. Since knowing about your pregnancy, how often have you taken multivitamins as recommended by your doctor or midwife?

| Never | Rarely | Sometimes | Often | Every day |
| --- | --- | --- | --- | --- |

1. How often do you (or did you) consume adequate amounts of calcium as recommended for pregnant women by your doctor or midwife?

| Never | Rarely | Sometimes | Almost daily | Every day |
| --- | --- | --- | --- | --- |

1. Since knowing about your pregnancy, how often have you consumed at least five fruits/vegetables a day?

| Never | Rarely | Sometimes | Almost daily | Every day |
| --- | --- | --- | --- | --- |

1. Since knowing about your pregnancy, how often have you consumed an adequate quantity of fiber? (Fiber sources include beans, nuts, bran cereal, wholegrain bread, etc.)

| Never | Rarely | Sometimes | Often | Every day |
| --- | --- | --- | --- | --- |

1. Since knowing about your pregnancy, how often have you smoked cigarettes?

| Never | Rarely | Sometimes | Often | Every day |
| --- | --- | --- | --- | --- |

1. Since knowing about your pregnancy, how often have you consumed alcoholic beverages (i.e., beer, wine and liquor)?

| Never | Rarely | Sometimes | Almost daily | Every day |
| --- | --- | --- | --- | --- |

1. How many weeks were you pregnant when you started seeing a doctor or midwife for prenatal care?

| 4 weeks | 8 weeks | 12 weeks | 20 weeks | More than 24 weeks |
| --- | --- | --- | --- | --- |

1. How often have you missed your prenatal care visits?

| Never | Rarely | Sometimes | Often | I have not attended prenatal care at all |
| --- | --- | --- | --- | --- |

**Thank you so much for your time and participation.**
